# Massive hemoptysis successfully treated with extracorporeal membrane oxygenation and endobronchial thrombolysis

**DOI:** 10.5935/0103-507X.20180002

**Published:** 2018

**Authors:** Antônio Aurélio de Paiva Fagundes Júnior, Renato Bueno Chaves, Amanda Robassini dos Santos, Humberto Alves de Oliveira, Marcello Henrique Paschoal

**Affiliations:** 1 Intensive Care Unit, Hospital Ortopédico e Medicina Especializada - Brasília (DF), Brazil.; 2 Intensive Care Unit, Instituto de Cardiologia do Distrito Federal - Brasília (DF), Brazil.; 3 Department of Thoracic Surgery, Hospital de Base do Distrito Federal - Brasília (DF), Brazil.

**Keywords:** Extracorporeal membrane oxygenation, Hemoptysis, Fibrinolytic agents, Case reports

## Abstract

Extracorporeal membrane oxygenation has been used to treat refractory hypoxemia in numerous clinical scenarios. The fundamental principles for the management of massive hemoptysis patients include protecting the airway and healthy lung, locating the source of bleeding and controlling the hemorrhage. We report the case of a patient with acute respiratory failure associated with massive hemoptysis secondary to lung laceration during cardiac surgery. The use of extracorporeal membrane oxygenation allowed patient survival. However, due to the great difficulty in managing pulmonary clots after hemoptysis, it was necessary to use an unusual therapy involving endobronchial infusion of a thrombolytic agent as described in rare cases in the literature.

## INTRODUCTION

In patients with severe acute respiratory failure, extracorporeal membrane oxygenation (ECMO) is a lifesaving strategy during lung injury recovery.^(1)^ However, descriptions of massive hemoptysis treated with a strategy using ECMO are very rare.^(2)^ Even more rare are reports of the use of endobronchial thrombolytics for clot removal in patients with hemoptysis. There is no report in the literature on the use of this associated endobronchial therapy in patients receiving ECMO.

## CASE REPORT

We describe the case of a 46-year-old female patient with rheumatic valvulopathy and a history of mitral and tricuspid valve repair 6 years prior who was hospitalized for heart failure associated with mitral and aortic insufficiency. The echocardiogram on admission revealed considerable dilated left ventricular cardiomyopathy (diastolic diameter: 65mm; systolic diameter: 42mm) with 68% ejection fraction and 49mmHg pulmonary artery systolic pressure. The examination revealed marked mitral insufficiency and moderate stenosis in addition to moderate aortic insufficiency and mild tricuspid insufficiency.

The patient underwent mitral and aortic valve replacement with mechanical prostheses. During surgery, she sustained iatrogenic laceration of the left lung followed by severe and refractory acute respiratory insufficiency associated with massive hemoptysis. Selective intubation was attempted but failed. The laceration was surgically corrected, but arteriography was not performed.

The patient developed refractory hypoxemia (partial pressure of oxygen [PaO_2_] of 54mmHg, oxygen saturation [SatO_2_] of 84% and fraction of inspired oxygen [FiO_2_] of 100%) and hypercapnia (partial pressure of carbon dioxide [PaCO_2_] of 109mmHg and pH 6.9). The intraoperative echocardiogram revealed preserved biventricular function, but the pulmonary artery systolic pressure was 62mmHg. Venovenous ECMO (VV-ECMO) was initiated during surgery. However, due to the associated severe hemodynamic instability (noradrenaline 1mcg/kg/minute, vasopressin 0.04u/minute and dobutamine 10mcg/kg/minute) and problems with the VV-ECMO flow, we opted for immediate change to venoarterial ECMO (VA-ECMO) (MAQUET^®^ Rotaflow RF 32 Console, Quadrox PLS System). A 17Fr cannula was inserted in the left femoral artery, and a 21Fr cannula was inserted in the right femoral vein. The latter was positioned in the right atrium and guided by echocardiography. Both cannulas were inserted during surgery and by dissection. An anterograde reperfusion cannula (6Fr) was inserted in the left superficial femoral artery by dissection. Invasive blood pressure was placed in the right radial artery. We programmed the VA-ECMO at 4,200rpm, 4L/minute flow, and 4L/minute sweep with 80% FiO_2_. The initial mechanical ventilation programming was performed in the pressure-controlled mode with positive end-expiratory (PEEP) of 10cmH_2_O, FiO_2_ of 25%, respiratory rate of 12irpm, controlled pressure of 15cmH_2_O, and plateau pressure of 25cmH_2_O, maintaining the tidal volume at 5mL/kg. Lactate was 4.8mmol/L at admission to the intensive care unit and 2.1mmol/L after 24 hours of ECMO.

**Figure 1:**
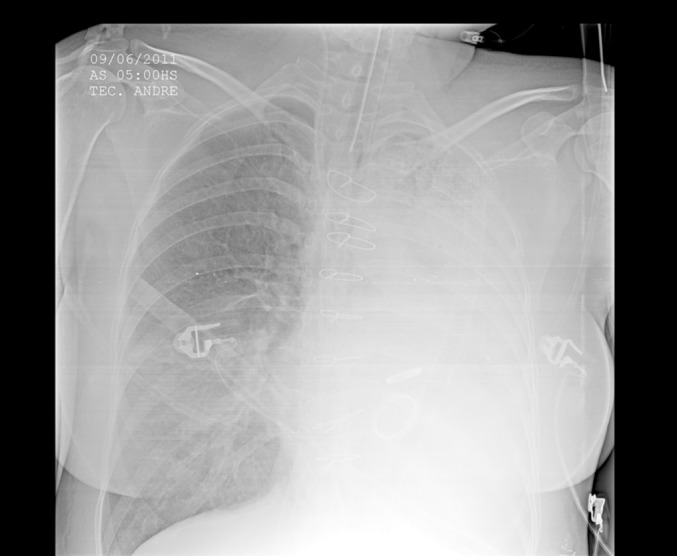
Chest X-ray at admission revealing extensive opacity in the entire left hemithorax, secondary to laceration of the left lung, associated with massive hemoptysis.

After surgery, the patient developed a large number of clots in the bronchial tree, which were difficult to remove due to the tree's dryness. Sequential bronchoscopies were performed on the first and second postoperative days in an attempt to remove the clots, but all were unsuccessful (Figure 1).

Forty-eight hours after VA-ECMO was installed and when hemoptysis was controlled safely, anticoagulation was started with intravenous unfractionated heparin (500 units per hour) to maintain an activated coagulation time of 150 - 180 seconds.

**Figure 2:**
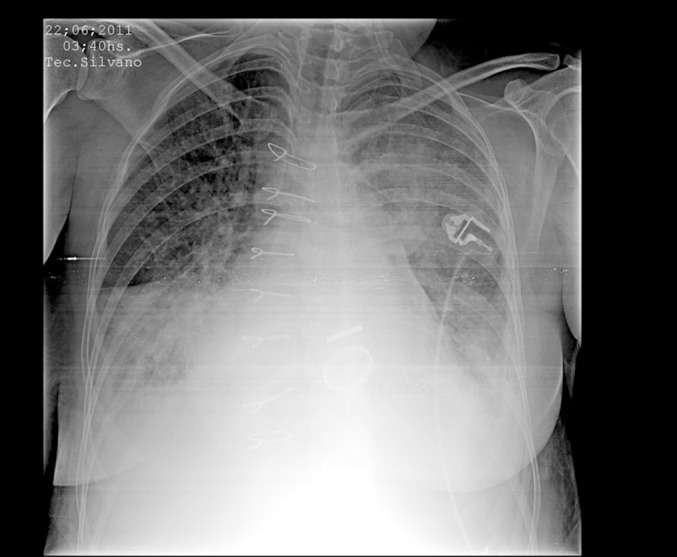
Chest X-ray revealing improvement of the radiological picture after endobronchial thrombolysis.

The patient presented progressive hemodynamic improvement with suspension of the vasoactive drugs on the second postoperative day. However, she persisted with a severe pulmonary condition and was dependent on ECMO without tolerating the FiO_2_ reduction in this device. On the third postoperative day, endobronchial streptokinase (30,000u/30mL) was infused with 10 mL infused every 15 minutes (total of three 10mL infusions) with subsequent removal of large clots. The process was repeated for three consecutive days, after which a significant improvement in the pulmonary picture was observed both from the radiological and gasometric points of view (Figure 2). It was possible to progressively reduce the ECMO FiO_2_ with maintenance of adequate PaO_2_ and SatO_2_ (PaO_2_/FiO_2_ ratio greater than 300 with ECMO FiO_2_ of 21% and FiO_2_ of 40% with mechanical ventilation). After four days of support, withdrawal of the ECMO was planned. The patient was sedated (Richmond Agitation and Sedation Scale [RASS]: 5) with midazolam and fentanyl without vasoactive drugs with a mean arterial pressure of 66mmHg, heart rate of 77bpm, arterial lactate of 1.1mmoL/L, central venous oxygen saturation (SVcO_2_) of 76% and bicarbonate of 24mEq/L, revealed by arterial gasometry, with a PaO_2_/FiO_2_ ratio of 305. The patient presented mechanical ventilation FiO_2_ of 40% and ECMO FiO2 of 21%. The patient maintained a good urine output with 1.0mg/dL creatinine and 70mg/dL urea.

An echocardiogram was performed at the bedside, and the ECMO support was reduced to 1,500rpm, corresponding to a flow of 1.3L/minute. In this condition, the examination revealed an estimated ejection fraction of 45%, aortic valve velocity-time integral of 14cm and systolic velocity (S-wave) of the mitral annulus of 9cm/s via tissue Doppler. Based on the clinical and echocardiographic data, the ECMO support was removed after 91 hours.

On the sixth postoperative day, antibiotic therapy was initiated due to focal sepsis in the lung. Vancomycin and meropenem were administered for 10 days with good clinical response. The requested cultures (blood cultures, uroculture and tracheal aspirate culture) did not identify microorganism growth.

Due to the progressive respiratory improvement, extubation was performed after 7 days of mechanical ventilation and 3 days after ECMO withdrawal. The patient developed left lower limb paresis with detection of peripheral nerve damage related to the ECMO arterial cannulation site.

**Figure 3:**
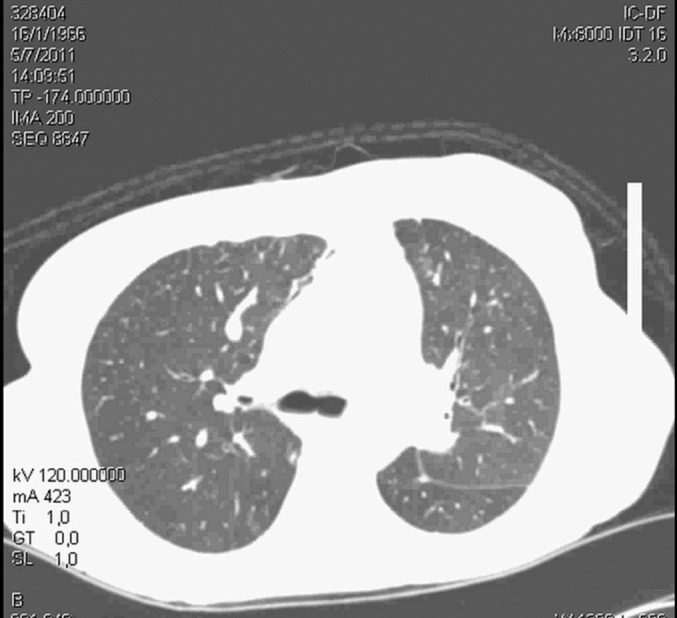
Chest computed tomography performed 25 days after surgery revealing recovery of the lung condition.

The patient presented good clinical evolution with complete recovery of the respiratory condition (Figure 3). She was discharged on the 30^th^ postoperative day with left lower limb paresis (grade 3 muscle strength by the Medical Research Council). The patient was followed up on an outpatient basis and resumed her usual activities with independence. She remained asymptomatic from a respiratory and cardiovascular point of view, maintaining outpatient use of 35 mg/week warfarin, 5mg/day enalapril and 12.5mg/day carvedilol. The control echocardiogram at 6 months after discharge revealed an ejection fraction of 52.82% with mechanical prosthesis in a normal mitral position, discrete mitral valve insufficiency, a maximum diastolic gradient of 14mmHg and a mean diastolic gradient of 5.5mmHg. The mechanical prosthesis in aortic position functioned normally. The maximum systolic gradient was 21mmHg, and the mean was 11 mmHg. Discrete left ventricular systolic dysfunction was noted with a slight increase in the left atrium and a moderate increase in the left ventricular diameter (diastolic diameter: 58mm; systolic diameter: 42mm). Discrete tricuspid insufficiency was also observed, and the pulmonary artery systolic pressure was estimated at 43mmHg.

**Figure 4:**
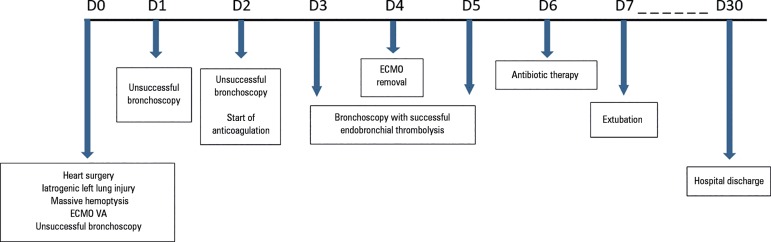
Timeline of the evolution of the case report. ECMO - extracorporeal membrane oxygenation; VA - venoarterial.

The patient was discharged from the valvulopathy outpatient clinic of the *Instituto de Cardiologia do Distrito Federal*, Brasília 6 months after hospital discharge to continue follow-up in her hometown in the state of Bahia (Figure 4).

## DISCUSSION

The use of ECMO in cases of severe and refractory acute respiratory failure has become the standard therapy worldwide.^(1)^ However, its use in cases of massive hemoptysis is rarely described in the literature.^(2)^

In the reported case, the only option that allowed the patient to survive an acute, very rapidly setting condition refractory to the initial measures was circulatory and respiratory assistance with VA-ECMO.

Because hemoptysis was controlled, it was possible to start anticoagulation with unfractionated heparin safely and without complications. Hemodynamic support through VA-ECMO combined with blood volume and blood loss correction and normalization of PaO_2_ and PaCO_2_ allowed the reduction and subsequent withdrawal of vasoactive drugs.

However, due to the large number of resected clots identified during the bronchoscopies performed, no improvement in the respiratory condition was noted on subsequent days. Thus, we decided to searched the PubMed database using the terms "massive hemoptysis and thrombolytic". The search resulted in 26 articles, including 3 rare cases^(4-6)^ successfully treated with thrombolytic therapy by infusion using a bronchoscope. The procedure was thus performed in a similar manner as described.

Streptokinase was diluted in 0.9% saline solution, yielding a 1000u/mL solution. Then, 10mL was infused. Next, bronchial lavage was performed with removal of the clots. We repeated the procedure thrice with a 10-minute interval between each infusion and subsequent lavage. The therapy was repeated daily for 3 consecutive days. The endobronchial administration of a thrombolytic agent allowed the clot to be removed effectively. This removal had previously not been possible, thus ensuring significant clinical improvement of the patient.

Significant radiological improvement was noted, as shown in the sequential images (Figures 1, 2 and 3). It was possible to withdraw ECMO after 91 hours. Extubation occurred after 7 days of ventilation with complete recovery of the lung condition demonstrated by chest tomography performed before hospital discharge.

The successful use of ECMO in patients with respiratory insufficiency secondary to hemoptysis is well described in the literature^(7-10)^ despite the high risk of bleeding after implantation due to the need for anticoagulation. However, this is the first case described in the literature documenting the use of endobronchial thrombolytic treatment in a patient receiving VA-ECMO support.

The *Instituto de Cardiologia do Distrito Federal* is a tertiary, philanthropic hospital and serves as a reference in the Federal District for cardiac surgeries through an agreement with the Brazilian Unified Health System (Sistema Único de Saúde - SUS). The institute has a cardiac surgery team and a full-time team of perfusionists as well as experience with VV- and VA-ECMO. This therapy is commonly used in the institution for the treatment of cardiogenic shock, ventricular failure after cardiotomy and graft failure after heart transplantation. In 2016, 48 cases of VA-ECMO were performed: 21 in adult patients and 17 in pediatric patients. Given the team's experience with ECMO implantation, it was possible to define the approach quickly, avoiding the development of multiple organ failure.

## CONCLUSION

We described the case of an intraoperative complication of cardiac surgery that led to severe acute respiratory failure and massive hemoptysis, necessitating the immediate establishment of venoarterial extracorporeal membrane oxygenation. Additionally, it was necessary to use a thrombolytic agent via the endobronchial route for removal of clots. This is a last resort therapy, and this was the first case described.
